# Supplementary material for: A universal opportunity model for human mobility
Source: Sci Rep. 2020 Mar 13;10:4657. doi: 10.1038/s41598-020-61613-y (PMC7070048; doi:10.1038/s41598-020-61613-y)
Supplement: Supplementary file 1 — Supplementary Information [file 41598_2020_61613_MOESM1_ESM.pdf]

Supplementary Information for
A universal opportunity model for human mobility

Er-Jian Liu, Xiao-Yong Yan

Contents

S1 The derivation of the radiation model **2**

S2 The derivation of the OPS model **2**

S3 The derivation of the OO model **3**

S4 Comparison among different models **4**

References **4**

S1 The derivation of the radiation model

The radiation model [S1] assumes that when seeking job offers, the commuter will choose the closest workplace to his/her home, whose benefit is higher than the best offer available in his/her home county, i.e., the benefit of home is higher than the benefits of the intervening opportunities and lower than the benefit of the workplace. According to the above assumption, the probability that location j is selected by the individual at location i is

$$Q_{ij} = \int_0^\infty \mathrm{Pr}_{m_i}(z)\mathrm{Pr}_{s_{ij}}(< z)\mathrm{Pr}_{m_j}(> z)\mathrm{d}z, \tag{S1}$$

where m_i is the number of opportunities at location i, m_j is the number of opportunities at location j, s_{ij} is the number of intervening opportunities [S2] (i.e., the sum of the number of opportunities at all locations whose distances from i are shorter than the distance from i to j), Pr_{m_i} is the probability that the maximum benefit obtained after m_i samplings is exactly z, $\mathrm{Pr}_{s_{ij}}(< z)$ is the probability that the maximum benefit obtained after s_{ij} samplings is less than z, $Pr_{m_j}(> z)$ is the probability that the maximum benefit obtained after m_j samplings is greater than z.

Since $\mathrm{Pr}_x(< z) = p(< z)^x$, we obtain

$$\mathrm{Pr}_x(z) = \frac{\mathrm{d}\mathrm{Pr}_x(< z)}{\mathrm{d}z} = xp(< z)^{x-1}\frac{\mathrm{d}p(< z)}{\mathrm{d}z}, \tag{S2}$$

$$\mathrm{Pr}_x(> z) = 1 - p(< z)^x. \tag{S3}$$

Eq. (S1) can be written as

$$\begin{aligned} Q_{ij} &= \int_0^\infty \mathrm{Pr}_{m_i}(z)\mathrm{Pr}_{s_{ij}}(< z)\mathrm{Pr}_{m_j}(> z)\mathrm{d}z \\ &= m_i \int_0^1 (p(< z)^{m_i+s_{ij}-1} - p(< z)^{m_i+s_{ij}+m_j-1})\mathrm{d}p(< z) \\ &= m_i(\frac{p(< z)^{m_i+s_{ij}}}{m_i + s_{ij}}\Big|_0^1 - \frac{p(< z)^{m_i+s_{ij}+m_j}}{m_i + s_{ij} + m_j}\Big|_0^1) \\ &= \frac{m_i m_j}{(m_i + s_{ij})(m_i + s_{ij} + m_j)}. \end{aligned} \tag{S4}$$

Then, the probability of the individual at location i choosing location j as the destination is

$$P_{ij} = \frac{Q_{ij}}{\sum\limits_j Q_{ij}} \propto \frac{m_i m_j}{(m_i + s_{ij})(m_i + s_{ij} + m_j)}. \tag{S5}$$

S2 The derivation of the OPS model

The OPS model [S3] assumes that the destination selected by the individual is the location that presents a higher benefit than the benefit of the origin and the benefits of

the intervening opportunities. According to the above assumption, the probability that location j is selected by the individual at location i is

$$Q_{ij} = \int_0^\infty \mathrm{Pr}_{m_i+s_{ij}}(z)\mathrm{Pr}_{m_j}(>z)\mathrm{d}z, \tag{S6}$$

where $\mathrm{Pr}_{m_i+s_{ij}}$ is the probability that the maximum benefit obtained after m_i+s_{ij} samplings is exactly z, the other variables have the same meaning as in Eq. (S1). According to Eqs. (S2-S3), Eq. (S6) can be written as

$$\begin{aligned} Q_{ij} &= \int_0^\infty \mathrm{Pr}_{m_i+s_{ij}}(z)\mathrm{Pr}_{m_j}(>z)\mathrm{d}z \\ &= (m_i+s_{ij})\int_0^1 [p(<z)^{m_i+s_{ij}-1} - p(<z)^{m_i+s_{ij}+m_j-1}]\mathrm{d}p(<z) \\ &= (m_i+s_{ij})\frac{p(<z)^{m_i+s_{ij}}}{m_i+s_{ij}}\Big|_0^1 - (m_i+s_{ij})\frac{p(<z)^{m_i+s_{ij}+m_j}}{m_i+s_{ij}+m_j}\Big|_0^1 \\ &= \frac{m_j}{m_i+s_{ij}+m_j} \\ &= \frac{m_j}{S_{ij}}. \end{aligned} \tag{S7}$$

Then, the probability of the individual at location i choosing location j as the destination is

$$P_{ij} = \frac{Q_{ij}}{\sum\limits_j Q_{ij}} \propto \frac{m_j}{S_{ij}}. \tag{S8}$$

S3 The derivation of the OO model

The OO model assumes that the individual chooses the location whose benefit is higher than the benefit of the origin. According to the above assumption, the probability that location j is selected by the individual at location i is

$$Q_{ij} = \int_0^\infty \mathrm{Pr}_{m_i}(z)\mathrm{Pr}_{m_j}(>z)\mathrm{d}z, \tag{S9}$$

the variables have the same meaning as in Eq. (S1). According to Eqs. (S2-S3), Eq. (S9) can be written as

$$\begin{aligned} Q_{ij} &= \int_0^\infty \mathrm{Pr}_{m_i}(z)\mathrm{Pr}_{m_j}(>z)\mathrm{d}z \\ &= m_i\int_0^1 (p(<z)^{m_i-1} - p(<z)^{m_i+m_j-1})\mathrm{d}p(<z) \\ &= m_i(\frac{p(<z)^{m_i}}{m_i}\Big|_0^1 - \frac{p(<z)^{m_i+m_j}}{m_i+m_j}\Big|_0^1) \\ &= \frac{m_j}{m_i+m_j}. \end{aligned} \tag{S10}$$

Then, the probability of the individual at location i choosing location j as the destination is

$$P_{ij} = \frac{Q_{ij}}{\sum_j Q_{ij}} \propto \frac{m_j}{m_i + m_j}. \tag{S11}$$

From equations S5, S8, S11, we can see that the radiation model, the OPS model and the OO model are all special cases of our UO model.

S4 Comparison among different models

A more detailed measure of a model's ability to predict mobility patterns can be implemented in terms of the travel fluxes between all pairs of locations produced by a model in comparison with real observations[S4]. We measure the fluxes predicted by different models in comparison with the real fluxes and find that the average fluxes predicted by the UO model are more in agreement with real observations than other three models for the fourteen data sets, as shown in Fig. S1-S3. Yet our UO model can accurately describe the individual's destination selection behavior at different spatiotemporal scales.

References

[S1] Simini, F., González, M. C., Maritan, A. & Barabási, A.-L. A universal model for mobility and migration patterns. *Nature* **484,** 96-100 (2012).

[S2] Stouffer, S. A. Intervening opportunities: A theory relating mobility and distance. *Am. Sociol. Rev.* **5,** 845-867 (1940).

[S3] Liu, E. & Yan, X. New parameter-free mobility model: opportunity priority selection model. *Physica A* **526,** 121023 (2019).

[S4] Yan, X.-Y., Wang, W.-X., Gao, Z.-Y. & Lai, Y.-C. Universal model of individual and population mobility on diverse spatial scales. *Nat. Commun.* **8,** 1639 (2017).

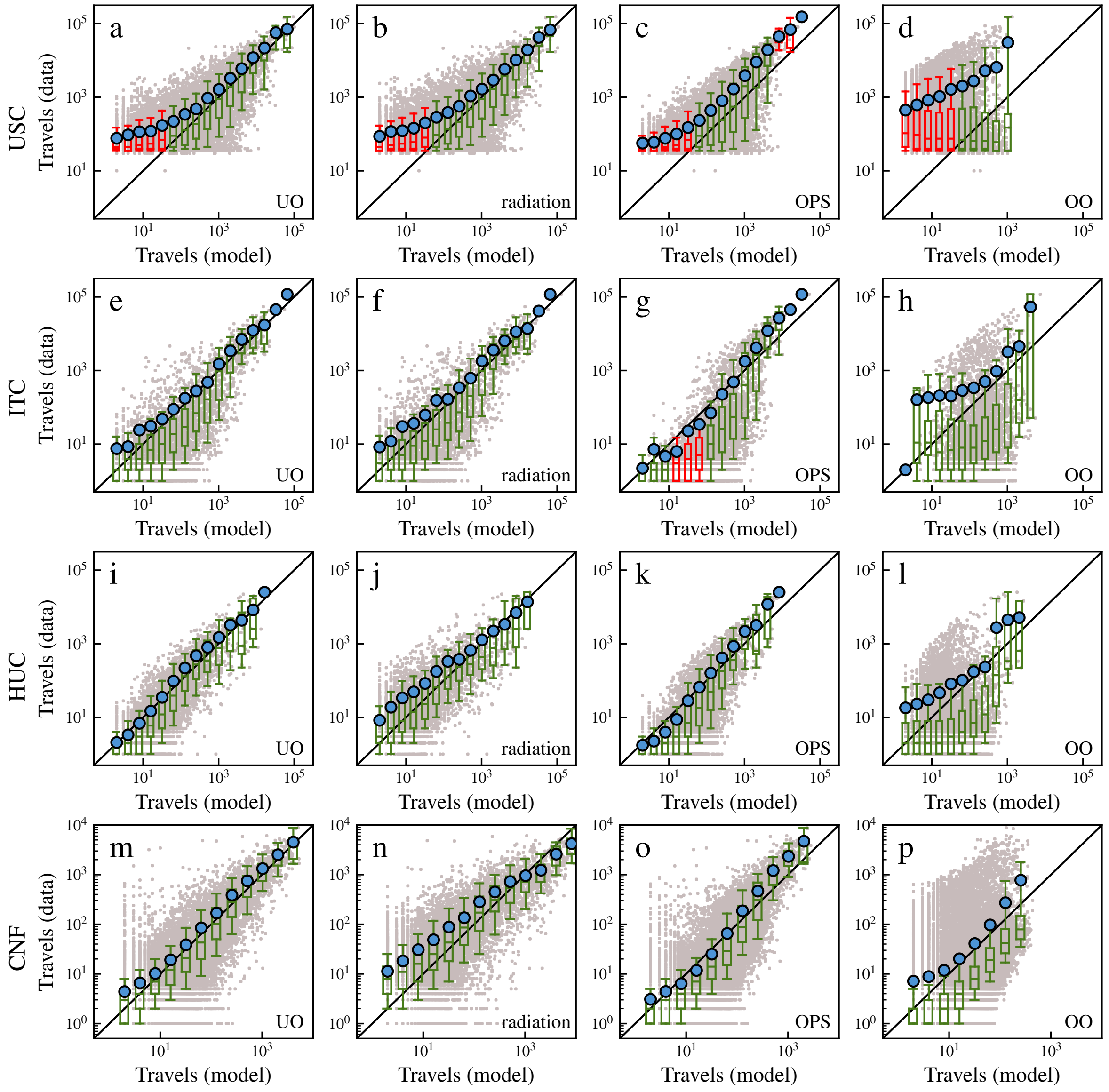

Figure S1: **Comparing the observed fluxes with the predicted fluxes for commuting and freight.** The grey points are scatter plot for each pair of locations. The blue points represent the average number of predicted travels in different bins. The box-plots, obtained via standard statistical methods,represent the distribution of the number of predicted travels in different bins of the number of observed travels. A box is marked in green if the line y = x lies between 10 % and 91% in that bin and in red otherwise.

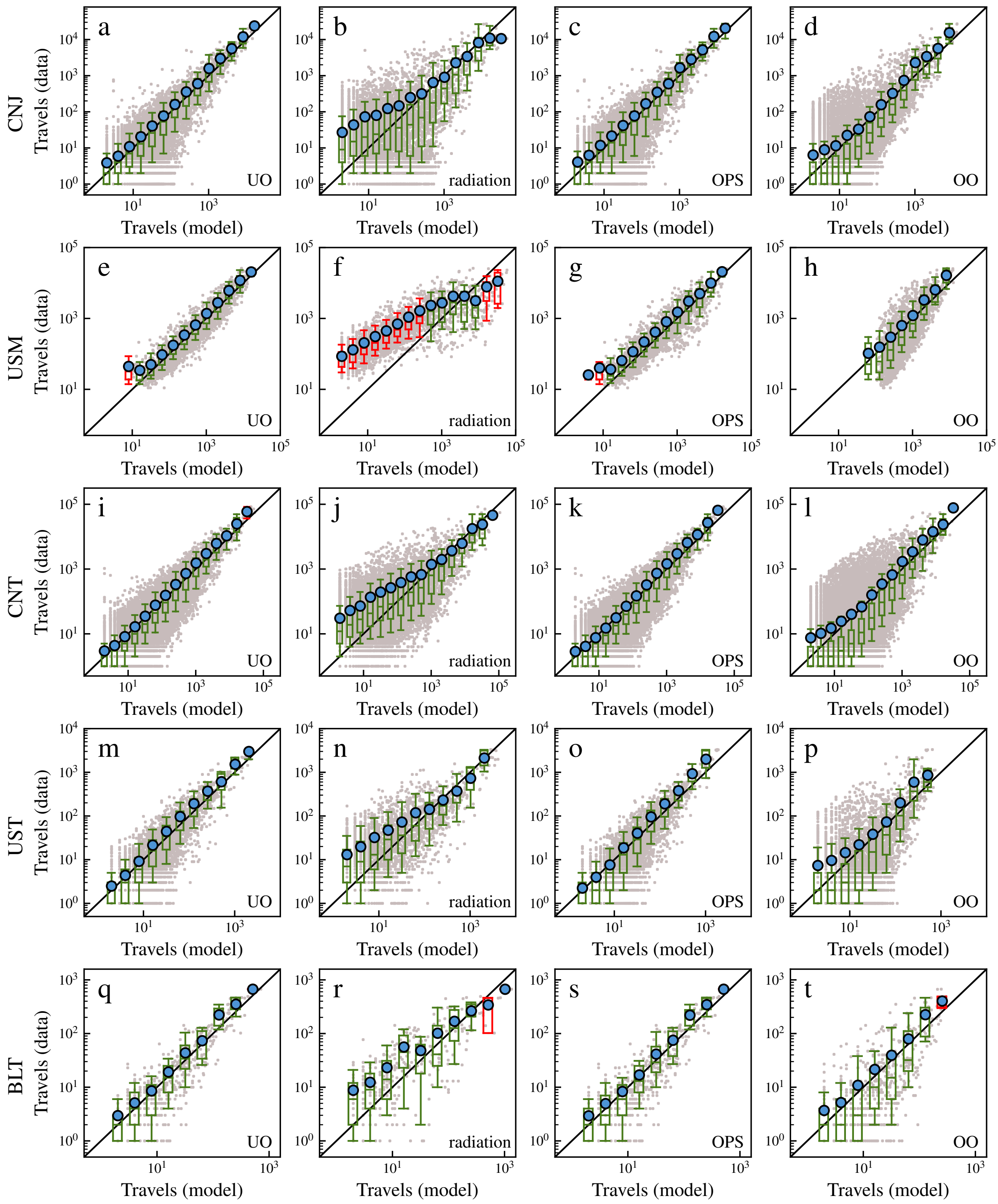

Figure S2: **Comparing the observed fluxes with the predicted fluxes for job hunting, migration and intercity travels.** Symbol meaning as in Fig. S1.

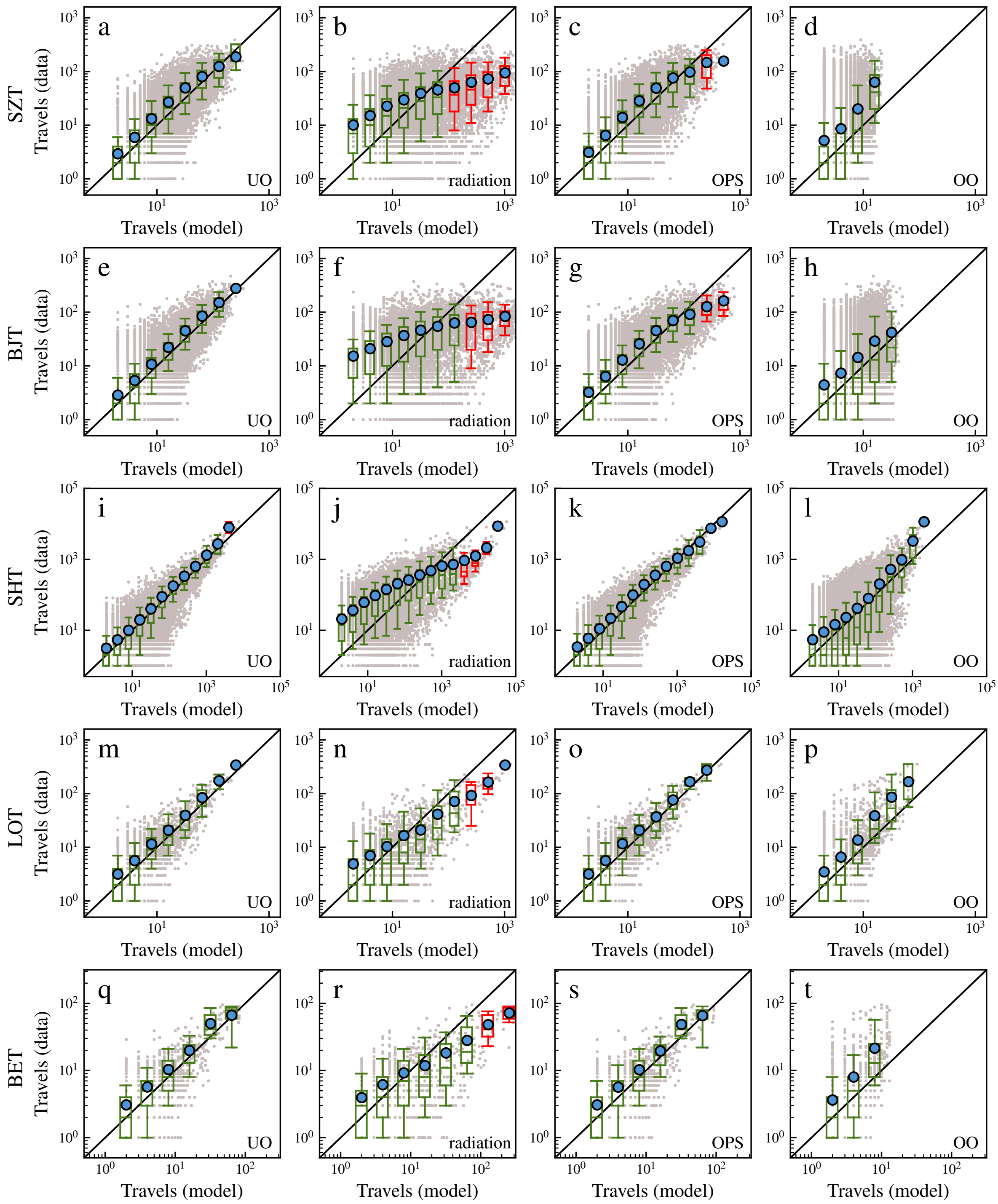

Figure S3: **Comparing the observed fluxes with the predicted fluxes for intracity trips.** Symbol meaning as in Fig. S1.
